# The Effects of Vitamin D Replacement with a High-Dose Treat-to-Goal Strategy

**DOI:** 10.3390/nu18030477

**Published:** 2026-02-01

**Authors:** Rodis D. Paparodis, Nikolaos Angelopoulos, Sarantis Livadas, Evangelos Karvounis, Dimitrios Askitis, Juan C. Jaume, Dimitrios T. Papadimitriou

**Affiliations:** 1 Hellenic Endocrine Network, 10563 Athens, Greece; n-angelopoulos@hen.gr (N.A.); s-livadas@hen.gr (S.L.); karvounis@endocrinesurgeon.gr (E.K.); dimitrios.askitis@gmail.com (D.A.); 2 Department of Medicine, Edward Hines Jr. VA Hospital, Hines, IL 60141, USA; juan.jaume@va.gov; 3 Division of Endocrinology, Diabetes and Metabolism, Department of Medicine, Loyola University Medical Center, Maywood, IL 60153, USA; 4 Division of Endocrinology, Diabetes and Metabolism, Department of Medicine, Athens Medical Center, 15125 Athens, Greece; 5 Center of Excellence in Endocrine Surgery, Euroclinic Hospital, 11521 Athens, Greece; 6 Pediatric Endocrine Clinics, 15125 Athens, Greece; 7 Faculty of Medicine, University of Thessaly, 41110 Larisa, Greece

**Keywords:** vitamin D deficiency, vitamin D replacement, high-dose supplementation

## Abstract

Introduction: Vitamin D deficiency [25(OH)D < 30 ng/mL] is widely prevalent globally and the efforts to tackle it have been rather unsuccessful to date. Despite different cutoffs used to define it, many clinicians adhere to the 2011 Endocrine Society definition. We present a special treat-to-target protocol aiming to restore and maintain vitamin D sufficiency. Methods: We reviewed the efficacy and safety of our vitamin D supplementation protocol over 5 years, and compared it to a group of patients who self-reported never taking vitamin D supplements. We recorded the baseline, 2-month, and annual 25(OH)D (D) measurements, along with subjects’ age, sex, BMI, history of osteoporosis, nephrolithiasis, nephrocalcinosis, and renal colics. According to our supplementation protocol, replenishment of vitamin D involves cholecalciferol dosing in two steps: a loading dose (LD) for 2 months and a maintenance dose (MD) thereafter. Please refer to the main text for loading and maintenance dose titration. Results: Of 8329 cases with vitamin D measurements, 2248 had adequate follow up data of 3524.5 patient-years and were included in the study: a total of 1575 intervention subjects and 673 controls, with an average follow-up of 18.8 months. Baseline vitamin D concentrations of 22.6 ng/mL (controls) did not change significantly (2 months: 22.2; 1 year: 21.7; 2 years: 22.0; 3 years: 23.8; 4 years: 21.8; and 5 years: 22.1 ng/mL), while concentrations of 21.9 ng/mL (intervention group) reached and remained 40 ng/mL (2 months: 41.0; 1 year: 39.4; 2 years: 39.0; 3 years: 39.3; 4 years: 40.4; and 5 years: 39.4 ng/mL). Vitamin D adequacy was achieved in 91.6% of patients in the intervention arm compared to only 16.9% in controls (*p* < 0.0001). Mean D and rates of adequacy were significantly higher over time in the intervention arm (*p* < 0.0001). The incidence of renal adverse events or hypervitaminosis did not differ between groups (*p* > 0.05). Conclusions: Our intervention protocol appears highly efficient in achieving and maintaining vitamin D adequacy over 5 years, with no increase in adverse events compared with controls, presenting it as an effective long-term strategy.

## 1. Introduction

Vitamin D is a steroid hormone synthesized from cholesterol, with the initial step occurring through the skin’s exposure to ultraviolet-B radiation from sunlight. Modern lifestyle is associated with minimal sun exposure, resulting in insufficient endogenous vitamin D production. Consequently, vitamin D deficiency, defined as a serum 25(OH)D concentration < 30 ng/mL, is highly prevalent in most countries, including sunny countries such as Greece [1,2,3,4,5]. Vitamin D deficiency has been linked to multiple adverse skeletal effects including secondary hyperparathyroidism, bone loss, and increased risk of fractures in older adults [6]. Moreover, it is associated with significant extra-skeletal effects on the immune system, including the risk and severity of infectious diseases [7,8]; metabolic abnormalities, including the risk for development of type 2 diabetes mellitus and disease control [9,10]; cardiovascular events and mortality [6,11]; and psychiatric disorders [12,13]. In adult patients at risk for vitamin D deficiency, including pregnant and lactating women, treatment with cholecalciferol or ergocalciferol supplements in doses up to 10,000 IU daily is recommended by the 2011 Endocrine Society Clinical Practice Guidelines Committee [14]. More specifically, recommendation 2.6 states that doses up to 4000 IU daily can be used for everyone above 8 years of age without medical supervision, while the goal is set to achieve a target concentration of 40–60 ng/mL [15]. Furthermore, the National and International Osteoporosis Foundations and the American Geriatric Society define vitamin D deficiency as a 25(OH)D concentration < 30 ng/mL [16]. In these guidelines, dosing limits and target serum levels are not well connected or related to the pre-existing degree of this nutrient’s deficiency. It is noteworthy that a complex interplay of adherence issues, interindividual variability, and seasonal effects negatively impacts both population-level and patient-specific vitamin D concentrations in most countries [17]. In fact, we have recently shown that most patients receiving low doses of vitamin D—even for long periods of time—remain deficient [18]. Moreover, few studies have assessed a specific treat-to-target strategy aiming to restore adequacy in a consistent, long-term mode. In a previously published work from our group, including 6912 subjects with vitamin D deficiency, patients on continuous treatment for more than 12 months with low (≤1200 IU daily), medium (1200–3000 IU daily), and high (>3000 IU daily) doses of vitamin D supplements achieved vitamin D adequacy in 42.3%, 55.5, and 68.0%, respectively. In search of a strategy that could combine safety, adherence, and effectiveness, we designed and instituted a replacement protocol meant to retain vitamin D adequacy during the entire year. The present study assesses the efficacy (vitamin D adequacy, i.e., 25-OH-D ≥ 30 ng/mL) and safety (risk of nephrolithiasis, renal colics, and hypervitaminosis D) of this replacement strategy using a retrospective, quality-control approach.

## 2. Methods

**Figure 1:**
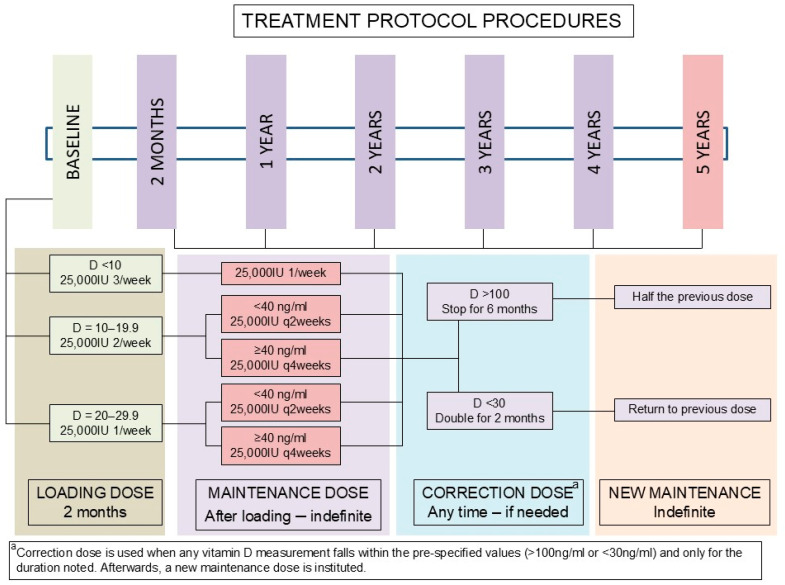
Treatment protocol procedures.

The Hellenic Endocrine Network is a national, private, not-for-profit research organization comprising Endocrinology, Diabetes, and Metabolism Clinics dispersed all over Greece, functioning as referral care sites for patients with disorders covering the entire spectrum of endocrinology. Vitamin D deficiency is managed independently in each clinic, as is standard worldwide. In 2017 though, a central algorithm was designed to facilitate treatment decisions, and it was subsequently followed by the present study’s participating clinics, including identical clinical record systems and follow-up schedules. The protocol was based on the prescribed medications available at that time in Greece, consisting of cholecalciferol 25,000 IU ampoules—Lecalcif® (SMB Technology SA, Marche en Famenne, Belgium, distributed by Rafarm Pharmaceuticals, Paeania, Greece) and Deltius® (Abiogen Pharma S.p.A, Pisa, Italy, distributed by ITF Pharmaceutical, Paleo Faliro, Greece)—sold as a packet of 4 ampoules each. The treatment protocol included a baseline measurement of 25(OH)D (vitamin D), followed by replacement in those patients found to be vitamin D-deficient [<30 ng/mL] in two steps; initially we used a loading dose (LD) aiming to restore sufficiency within 2 months. The LD was dependent on the baseline vitamin D concentration (Figure 1 and Supplementary Table S1). A second vitamin D measurement was performed after 2 months, based on which the maintenance dose was determined. The MD was continued until further notice if there was no hypervitaminosis D (vitamin D > 150 ng/mL) or hypovitaminosis D (vitamin D < 30 ng/mL), with dose calibration performed yearly when patients attended the clinic during their endocrine follow-up visits. The rationale of the present protocol was to rapidly replenish deficient levels, ensuring optimal serum concentrations for bone [6] and other potential health benefits [7,8,9,10,11,12,13,19,20], while follow-up with a long-term maintenance dose and yearly measurements would allow individualized adjustments to sustain optimal levels while preventing toxicity or under-supplementation. The dose of our vitamin D supplements falls well within the reference provided by the 2011 Endocrine Society’s Clinical Practice Guideline for patients at risk for vitamin D deficiency [14]. Vitamin D treatment was offered to all clinic patients with vitamin D deficiency after discussing benefits and potential harm. Those who followed our clinical protocol constitute this study’s intervention population, while patients who opted not to receive any vitamin D supplements or received small quantities without medical supervision (≤1000 IU daily for a period of up to 2 months) served as controls. The latter were included in the control group, because they are known to achieve minimal rates of vitamin D adequacy (28.2% when using <1200 IU daily for up to 12 months), based on our prior studies, and are not expected to affect treatment outcomes [18]. Vitamin D measurements were performed in certified clinical laboratories using Chemiluminescent Immunoassays (CLIA)—most often used in clinical laboratories worldwide (Abbott Architect, DiaSorin Liaison, Roche Elecsys, Siemens ADVIA).

### 2.1. Data Recording

Data were collected retrospectively from four clinics of the Hellenic Endocrine Network in a quality-control format to evaluate the efficacy and safety of this replacement strategy using a data collection form. Patient age on study enrollment, sex, history of osteoporosis, nephrolithiasis/nephrocalcinosis, height, and weight at the introduction of vitamin D, and whether they received supplementation or not (control population) were recorded. In all patients, the baseline body mass index (BMI) was calculated using the National Heart, Lung and Blood Institute formula:—BMI (kg/m^2^) = weight (kg)/height^2^ (m)^2^—and they were categorized based on the World Health Organization recommendations for the Caucasian population. Vitamin D was measured at baseline, 2 months, 1, 2, 3, 4, and 5 years. Data were included if patients had their annual visits within 2 months from the previous visit anniversary. Missing data imputation was not used. Information on renal calculi or colics, and any available imaging report aimed at the kidneys was recorded during all patient visits and reviewed for the present analysis.

### 2.2. Treatment Protocol

The treatment protocol is shown in Figure 1. Patients with vitamin D deficiency (<30 ng/mL) were split into three loading-dose treatment groups, dependent on their baseline vitamin D concentration: for those with serum vitamin D < 10 ng/mL, treatment was initiated with three 25,000 IU ampules per week in split doses; for those with baseline serum vitamin D between 10.0 and 19.9 ng/mL, the loading dose was 2 ampoules per week in split doses; and for those with baseline serum vitamin D between 20.0 and 29.9 ng/mL, the loading dose was 1 ampoule per week. The days and timing of vitamin D administration were left to the patients’ discretion.

After 2 months of LD, a new measurement of vitamin D was performed, and patients continued receiving an MD indefinitely if vitamin D adequacy was achieved. For those with baseline serum vitamin D < 10 ng/mL, the MD was an ampoule weekly; for those with baseline serum vitamin D between 10.0 and 19.9 ng/mL and 20.0–29.9 ng/mL, the MD was an ampoule every 2 weeks if the attained vitamin D concentration was 30.0–39.9 ng/mL or an ampoule every 4 weeks if the attained vitamin D concentration was ≥40.0 ng/mL. In cases where vitamin D was deficient (<30 ng/mL), a correction was given for 2 months by doubling the MD, then returning to the usual MD, without retesting for vitamin D. In cases where vitamin D was over-replaced (>100 ng/mL), treatment was discontinued for 6 months, followed by a return to the usual MD, without retesting for vitamin D. The decision to avoid retesting in these cases aimed to prevent patient burden and medical care expenses and was based on the knowledge that such doses have not been associated with risks of vitamin D toxicity when used for only two months (dose increments). Discontinuation of all supplements for 6 months provides adequate time for vitamin D concentrations to return to the deficiency range, or the lowest end of adequacy, especially given that such doses have been recommended for long-term use in patients at risk for vitamin D deficiency (not those with a known deficiency) [14].

### 2.3. Outcomes

The primary outcome of this study was the treatment efficacy, estimated by comparison of the sufficiency rates and the mean vitamin D concentration between the intervention and control groups during the entire study period at each time point.

The secondary outcome was the safety of the treatment strategy, assessed by comparing the incidence rates of nephrolithiasis (defined as the identification of a new kidney stone on abdominal/retroperitoneal imaging performed for any indication), renal colics (in subjects who had no such events in the past), and any degree of nephrocalcinosis (in subjects who had no such occurrences in the past) between the intervention and control groups.

Differences in the incidence rates of a combined outcome comprising all of above, as well as differences in the incidence rates of hypervitaminosis D (defined as the number of measurements of 25-OH-vitamin D > 150 ng/mL), were also calculated, in case a low numbers of events tended to mask a statistically significant trend. The outcome assessors were blinded to group allocation during data abstraction to reduce information bias. The threshold we used for defining toxicity is based on the 2011 Endocrine Society Clinical Practice Guidelines 14, but this is not universally accepted as such: some studies suggest that serum 25-OH vitamin D concentrations have to surpass 240 ng/mL before 1,25-(OH)2-vitamin D production increases [21], while others suggest that 25-OH vitamin D concentrations above 80 ng/mL could produce toxicity in some individuals [22].

### 2.4. Exclusion Criteria

All minors (<18 years) were excluded from the study, as well as those who followed the protocol regimen inconsistently (intake < 80% of the prescribed medications, based on self-reports and filled electronic prescriptions, as documented in the medical records), those who received treatment for <2 months, those who did not undergo regular follow-up vitamin D measurements, those who did not undergo vitamin D measurement within 15 days of the initial 2 month time point or within 2 months from the yearly time points, those opting to receive other, non-prescribed treatments for vitamin D replacement, and those in whom the replacement regimen was altered in a way non-concordant to the treatment algorithm. Additionally, we excluded patients who had previously undergone bariatric surgery or other gastrointestinal resection for any indication, patients with known malabsorption syndromes, and patients using antiepileptic medications. The exclusion criteria were applied prior to patients’ group allocation.

### 2.5. Statistical Analysis

Statistical analysis and graphics generation were performed using GraphPad Prism v5.0 (GraphPad Software, Boston, MA, USA). Categorical data were compared using Fischer’s exact test or chi squared test. Odds ratios and 95% confidence intervals were estimated. Continuous variables were assessed for normality using the Kolmogorov–Smirnov test. The means of data following the normal distribution were compared with Student’s *t*-test or one-way ANOVA, while data not following normal distribution were compared with the non-parametric Mann–Whitney, Kruskal–Wallis, and Friedman tests; specific post-tests for multiple comparisons were also performed, where applicable. Per protocol analysis was performed, while intention-to-treat analysis was not performed, given the study exclusions. *p* values < 0.05 were deemed significant.

### 2.6. Ethical Approval

The present study was reviewed and approved by the Institutional Review Board of the Hellenic Endocrine Network (approval number Ν2024/0121317, date of expiration 31 December 2029). Given the retrospective, quality-control, and anonymized nature of this study and the minimal risks to our patients, an exemption from the requirement for informed consent was granted by the IRB. The study protocol is in agreement with the 1964 Declaration of Helsinki and its subsequent modifications, including the 75th WMA General Assembly, held in Helsinki, Finland, in October 2024.

## 3. Results

**Figure 2:**
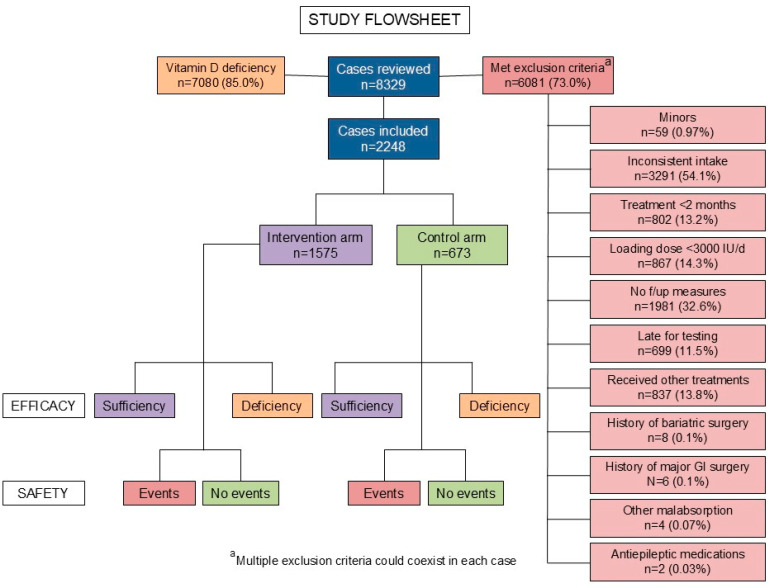
Study flowsheet.

We reviewed the charts of n = 8329 consecutive patients who had 23,124 vitamin D measurements in our records. Out of these, n = 7080 (85.0%) had vitamin D deficiency at baseline, and n = 2248 had follow-up measurements that met our inclusion criteria, and they were enrolled in the present study. The exclusion indications for the remaining subjects are depicted in Figure 2, which contains the entire study flowsheet. The treatment protocol and the dose adjustments effectuated are depicted in Figure 1. The baseline features of the entire study population and the intervention and control subjects separately, as well as their comparisons, are presented in Table 1. Of note, the intervention subjects were older and more commonly had a pre-existing diagnosis of osteoporosis or osteopenia (*p* < 0.001).

Overall, n = 1575 subjects followed the treatment plan (intervention group), and n = 673 did not follow our recommendations; they never received vitamin D replacement in a systematic way and, thus, they formed our control group. The follow-up duration ranged from 2 months to 5 years, totaling 42,294 patient-months or 3524.5 patient-years. The mean follow-up duration of the entire study population was 18.8 months; the intervention subjects had a mean follow-up of 19.5 months, and control subjects had a mean follow-up of 17.2 months. Overall, 5541 vitamin D measurements during follow-up were retrieved and included in the study; n = 3987 in the intervention group, and n = 1554 in the control group.

**Figure 3:**
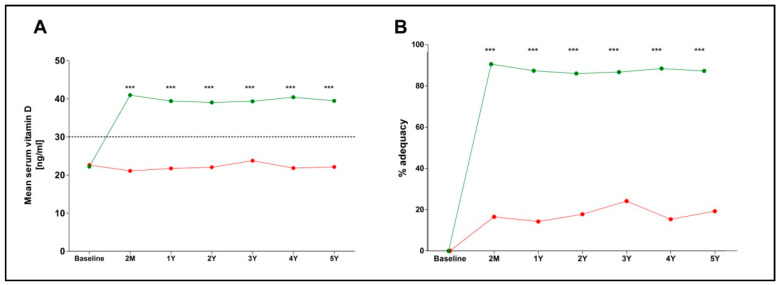
Comparison between the vitamin D restoration efficacy of our proposed treatment protocol and controls. (**A**) Mean serum vitamin D over time. The dashed line represents the serum concentration of vitamin D defined as adequate (30 ng/mL) (**B**) Rates of vitamin D adequacy over time. The green line represents the intervention group, while the red line represents the control group. ***: *p* < 0.001 when Intervention and Control groups were compared.

The primary outcome was highly statistically significant in all three metrics. Vitamin D adequacy was identified in n = 3654/3987 (91.6%) of the measurements in the intervention group and n = 263/1554 (16.9%) in the control group, and their difference was highly statistically significant, *p* < 0.0001, yielding an OR of 53.9, (95% CI 45.3–65.1) and a relative risk of 5.4 (95% CI 4.8–6.0). The mean vitamin D concentration and the rates of adequacy for each group are shown in Table 2 and Figure 3; both these measurements were statistically significantly higher in the intervention arm compared to controls at all time points (*p* < 0.001).

The secondary outcomes data are presented in Table 3; in brief, a new diagnosis of nephrolithiasis, nephrocalcinosis/microlithiasis, renal colic, their combination, or hypervitaminosis with vitamin D was not statistically significantly different between groups (*p* > 0.05) over a 5-year period.

In an ad hoc analysis, all vitamin D measurements < 30 ng/mL in the intervention arm were reviewed. Out of these, the following numbers of subjects had discontinued their treatment prior to the measurement, without notifying the treating physician: n = 6/95 (6.3%) at 2 months, n = 53/84 (63.1%) at 1 year, 44/67 (65.7%) at 2 years, 31/44 (70.5%) at 3 years, n = 17/24 (70.8%) at 4 years, and n = 16/19 (84.2%) at 5 years.

A second ad hoc analysis was performed to estimate the rates of adequacy of vitamin D if a threshold of 20 ng/mL was used in the intervention population. In that scenario, treatment success was observed in 3953/3987 (99.1%) measurements, while treatment failure occurred in only 9/1544 (0.6%) patients at 2 months, 6/894 (0.7%) at 1 year, 7/621 (1.1%) at 2 years, 8/436 (1.8%) at 3 years, 1/290 (0.3%) at 4 years, and 3/200 (1.5%) at 5 years.

## 4. Discussion

The present work is a retrospective quality-control study aiming to assess the efficacy and safety of a simple, treat-to-target vitamin D deficiency treatment protocol instituted in four endocrinology clinics in Greece over 5 years. Our protocol consisted of a high-loading-dose replacement strategy, including once-, twice-, or thrice-weekly plans for two months, depending on the baseline serum vitamin D concentrations in our patients. This was followed by a graded reduction in dose that was continued indefinitely, with yearly measurements and dose adjustments when needed. The efficacy and safety of this regimen were compared between the treated patients and a group of vitamin D-deficient individuals, who did not follow any strict replacement strategy, usually did not take any vitamin D, but had follow-up vitamin D measurements in our clinics. Our strategy achieved high rates of vitamin D adequacy (≥30 ng/mL) with minimal occurrences of hypervitaminosis D, while the safety data did not suggest an increased risk of nephrolithiasis or renal colics.

Our study is the first to our knowledge to test a novel treat-to-target strategy for vitamin D replacement in a large real-world patient population. This was attained with high weekly doses and yearly adjustments, and the safety and efficacy were high, suggesting that this method that could be used to treat patients requiring vitamin D replacement. Its novelty is based on its structured, goal-driven protocol with predefined biochemical targets and mandatory reassessment, rather than the use of high or loading doses per se. In contrast to prior studies that employed fixed high-dose or loading-dose regimens without systematic dose recalibration, our strategy is distinguished by the following: (i) baseline 25(OH)D-stratified loading doses, (ii) mandatory reassessment at 2 months, (iii) explicit biochemical targets (25(OH)D ≥ 30 ng/mL) with dose de-escalation at ≥40 ng/mL, and (iv) long-term maintenance with annual monitoring and protocolized dose adjustments for up to 5 years. Previous high-dose or loading-dose regimens have generally focused on short-term repletion or fixed dosing schemes [23,24] and have not incorporated sustained, algorithm-based titration aimed at maintaining long-term adequacy. The treatment protocol was well accepted by our patients, including data from both sexes and all age groups. Baseline behavioral and motivational differences between treated patients and controls may partially explain the observed efficacy.

Our findings are in agreement with our previously published data [18], which revealed that patients who were treated with high doses (>3000 IU average daily dose) for long periods (>12 months consecutively) were those who achieved vitamin D adequacy. In that study, we reviewed the effects of vitamin D replacement strategies instituted by others in patients who visited our clinics for unrelated conditions. In fact, these doses were lower than the ones proposed in this work, and they were not dependent on the patients’ original vitamin D measurements, but rather a “one size fits all” strategy was used. Therefore, it is unsurprising that the maximal adequacy rate achieved in that study, with the highest doses for the longest periods of time, was approximately 60%, while our more aggressive and targeted strategy achieved a 91% overall success rate without an increase in adverse effects.

In general, vitamin D deficiency is defined as a serum 25-OH-vitamin D measurement < 30 ng/mL. According to multiple population-based and interventional studies, vitamin D deficiency is associated with poor health outcomes in bone health [25] and other outcomes, such as infections [26], metabolic syndrome [27], autoimmune diseases [28] and cancer [29], which become more prominent with increasing vitamin D deficits [30,31]. At the lowest end of the spectrum, when vitamin D concentrations are <15 ng/mL (or <12 ng/mL or <10 ng/mL according to other studies), patients also have decreased life expectancy [32]. Given that life has changed over the past several decades and sun exposure is limited, it comes as no surprise that vitamin D deficiency prevalence rose significantly in many populations, even in countries with many sunny days, such as Greece [2,5,18,33], or in desert countries of the Arab region [34]. Although significant controversy exists in the medical literature regarding when, whom, and how to treat this condition, a landmark Clinical Practice Guideline—the Endocrine Society’s 2011 Guideline—continues to be followed by large numbers of physicians worldwide [14]. According to that, adult patients at risk for vitamin D deficiency or known to be vitamin D-deficient could be treated with cholecalciferol or ergocalciferol doses averaging up to 10,000 IU daily. Other clinical practice guidelines recommend treatment of vitamin D-deficient individuals with a goal of adequacy (>30 ng/mL) only in specific populations, such as those with osteoporosis [35,36].

Each institution and virtually every endocrinologist has different protocols in place to address this condition, depending on their judgment or the clinical significance of vitamin D deficiency, while the treatment strategy in each case largely depends on these factors. And yet, we need to acknowledge that vitamin D sufficiency is not ensured for everyone with vitamin D > 30 ng/mL, as parathyroid hormone levels and calcium intake also need to be taken into account, as recently shown [37]. Our institution designed a treatment algorithm based on the only available prescription vitamin D supplements in Greece at that time in order to assist our patients who want to achieve and maintain vitamin D adequacy. This protocol consisted of two steps: an initial loading dose and a follow-up maintenance dose. The loading dose was administered over 2 months, with the aim to rapidly replenish vitamin D stores in the body, and its quantity was based on the baseline serum vitamin D concentration of our patients (Supplementary Table S1). This loading dose strategy led to substantial improvements in vitamin D concentrations, and most patients achieved adequacy, without evidence of hypervitaminosis D.

This finding was replicated at all time points and up to 5 years of treatment. The adequacy achieved by our control group was much lower and comparable to that observed in the general untreated patient population of Greece in our previous studies. Given that the treatment doses employed in our protocol erred towards the upper tier of the 2011 Endocrine Society’s Clinical Practice Guideline’s recommendation [14], but not including daily dosing, which has several benefits for some groups [38], we assessed any potential risks of hypervitaminosis or renal vitamin D toxicity. Both these safety outcomes were similar to those observed in controls, suggesting that our treatment protocol is both effective and safe. Given the retrospective design and the absence of randomization, the study confirms the protocol’s effectiveness in achieving and maintaining biochemical vitamin D adequacy, but causal inferences regarding clinical endpoints and superiority over other regimens require prospective, randomized evaluation.

### Study Limitations

Our study is limited by several factors that need to be taken into consideration: firstly, it is a retrospective real-world study, and this alone can introduce several forms of bias. The medications used were medically prescribed pharmaceutical vitamin D supplements; therefore, patients without medical insurance covering their prescriptions are less well represented in this work, even though in Greece, state family physicians are allowed to prescribe all medications to uninsured patients for free. Also, our definition of patients’ adherence to the proposed treatment is imperfect, based on medical records only, especially given the fact that the days and timing of medication intake was left entirely to our patients’ discretion. Despite that, the success rate of our strategy implies that these records held true, especially given the well-known vitamin D trends in the Greek population, published by our group and others in the past [2,5,18,33]. Of course, these effects cannot be extrapolated to patients with a history of gastrointestinal surgeries leading to malabsorption or other malabsorptive conditions, since those were excluded from our study. Future research will focus specifically on clinical factors affecting the changes in vitamin D concentration and how they can be used to alter the loading or maintenance dose effectively in order to further optimize this strategy. Moreover, the homogeneity of the study population, limited to a single national cohort, may impact the external validity of the findings, and further studies with more diverse populations are warranted to confirm these results. Furthermore, access to private practice physicians is somewhat limited to ethnic minorities residing in Greece due to higher likelihood of financial constraints, rendering our results non-generalizable to other ethnic/racial groups in Greece. Another source of potential bias pertains to safety, where patients who developed kidney stones could have changed providers, or kidney stones might have been missed if clinically silent, especially since not all patients underwent renal imaging during our clinical care. Despite this limitation, approximately 23% of our patients did undergo renal imaging for other clinical indications, and these records were reviewed by our study group, allowing for some degree of certainty regarding our findings. Additionally, vitamin D measurements were not performed in the same laboratory for all patients, even though the measurement methods were well validated and standardized, and the measurements were performed serially in the same lab for each patient, with all laboratories certified and subject to annual quality controls by external reviewers. Another limitation pertains to the fact that this study used intermittent high doses of vitamin D supplements, and the hereby proven safety and efficacy cannot be extrapolated to regimens involving smaller daily doses of equal or even higher potency, even though such regimens enhance adherence and reduce the patients’ pill burden [39]. Similarly, no fracture data was available in this line of work (although we hope to provide these in the future), thus preventing any assumptions regarding the regimen’s efficacy or safety in treating osteoporosis or preventing fractures. Equally important are the off-target effects of vitamin D, which is known to act on the immune system and psychiatric health, but no data on infections, autoimmunity, or psychiatric disorders are available either. Also, the measurements of serum 25-OH-vitamin D were not all performed during the same time of the year, which could affect the results of our study, given the well-known seasonal variability in vitamin D levels and deficiency in the Greek population [18]. Also, vitamin D treatment for serum 25(OH)D concentrations < 30 ng/mL is not the standard of care in all clinical practices globally, as some scholars consider a serum level > 20 ng/mL to be adequate. To facilitate data interpretation for colleagues targeting that threshold, we included the adequacy rates achieved with our regimen, which showed a 99% success rate. Finally, the study’s contribution to this field is limited in its current form, as it does not demonstrate that this protocol benefits patients with specific diseases.

## 5. Conclusions

Vitamin D deficiency is extremely common globally, and its impact appears significant. However, practical, scalable treatment strategies, capable of restoring adequacy in the majority of patients are still lacking. The present protocol is a simple, treat-to-target approach with long-term follow-up and appears promising, as it can replenish vitamin D stores without risks of toxicity in the vast majority of our patients. Nonetheless, these data arise from a retrospective real-world analysis. Further prospective randomized studies—or studies with parallel arms—aiming not only to restore adequacy but also to assess effects on clinically meaningful outcomes are needed to further evaluate this strategy.

## Figures and Tables

**Table 1:** Baseline features of the entire study population and each subgroup separately, along with their comparison.

	Total	INT	CON	*p* Value
Number of subjects	2248	1575	673	.
Mean age in years	45.0	45.4	44.4	0.008
SD	23.2	16.1	31.7	
Female Sex, n	1702	1230	472	<0.001
%	75.7	78.1	70.1	
Mean BMI	27.9	27.8	28.0	0.52
SD	6.5	6.4	6.7	
Serum vitamin D, mean	21.5	21.1	22.6	<0.001
SD	8.6	8.2	9.3	
Nephrolithiasis—n	245	172	73	0.98
%	10.9	10.9	10.8	
Osteoporosis—n	181	157	24	<0.001
%	.	10.0	3.6	
Osteopenia—n	473	408	65	<0.001
%	.	25.9	9.7	

BMI: body mass index, calculated as weight/height^2^ and measured in Kg/m^2^; serum vitamin D is measured in ng/mL (ref 30–100); SD: standard deviation. Nephrolithiasis is defined as a diagnosis based on clinical history obtained by our patients or confirmed by imaging during the study period. Osteoporosis is defined as a DEXA measurement of bone mineral density with a T-score < −2.4 at any site prior to the treatment allocation. Osteopenia is defined as a DEXA measurement of bone mineral density with a T-score between −1.0 and −2.4 at any site prior to the treatment allocation.

**Table 2:** Efficacy analysis: mean vitamin D measurements during the study period, rates of vitamin D adequacy of the two groups, and their comparison.

	Time Point	Baseline	2m	1y	2y	3y	4y	5y
CONTROLS	Mean 25-OH-D	22.6 ^b^	22.2	21.7	22.0	23.8	21.8	22.1
	SD	9.3	9.3	8.5	9.2	9.4	9.3	9.4
	Total n	673	642	342	198	162	137	73
	Adequacy n	0	106	48	35	39	21	14
	Adequacy %	0.0	16.5	14.2	17.7	24.1	15.3	19.2
INTERVENTION	Mean 25-OH-D	21.1	41.0 ^a^	39.4 ^a^	39.0 ^a^	39.3 ^a^	40.4 ^a^	39.4 ^a^
	SD	8.2	10.3	8.7	8.6	8.8	8.7	8.3
	Total n	1575	1546	894	621	436	290	200
	Adequacy n	0	1451	810	554	392	266	181
	Adequacy %	0.0	93.9 ^a^	90.6 ^a^	89.2 ^a^	89.9 ^a^	91.7 ^a^	90.5 ^a^
	*p* value	.	<0.0001	<0.0001	<0.0001	<0.0001	<0.0001	<0.0001

Serum vitamin D is measured in ng/mL (ref 30–100); SD: standard deviation. Adequacy is defined as a measurement of serum vitamin D between 30 and 100 ng/mL; 2m: 2-month time point; 1y: 1-year time point; 2y: 2-year time point; 3y: 3-year time point; 4y: 4-year time point; 5y: 5-year time point; ^a^: statistically significantly higher compared to the respective value of the control group; ^b^: statistically significantly higher compared to the respective value of the intervention group.

**Table 3:** Safety analysis: occurrence of new episodes of nephrolithiasis or a composite outcome of nephrolithiasis, nephrocalcinosis, and/or renal colics or elevated serum vitamin D concentration (>100ng/mL).

	Nephrolithiasis	Nephrocalcinosis/Microlithiasis	Renal Colic	Composite Outcome	Hypervitaminosis
Total n (%)	5/2248 (0.2%)	12/2248 (0.5%)	25/2248 (1.2%)	42/2248 (1.9%)	4/2248 (0.2%)
Control group n (%)	2/673 (0.3%)	5/673 (0.7%)	10/673 (1.5%)	17/673 (2.5%)	1/673 (0.1%)
Intervention group n (%)	3/1575 (0.2%)	7/1575 (0.4%)	15/1575 (1.0%)	25/1575 (1.6%)	3/1575 (0.2%)
*p* value	1.00	0.57	0.38	0.25	0.74
OR (95% CI)	0.64 (0.11–3.84)	0.60 (0.19–1.89)	0.64 (0.28–1.43)	0.62 (0.33–1.16)	1.28 (0.13–12.36)

Hypervitaminosis D: a serum 25-OH-D measurement > 100 ng/mL; OR: odds ratio; CI: confidence interval; composite outcome: a combined outcome comprising either the occurrence of a renal colic confirmed by a physician or a new diagnosis of nephrolithiasis or nephrocalcinosis/microlithiasis, visualized in any imaging modality (ultrasound, CT scan, or MRI).

## Data Availability

The research data of the study are available at the data repository of the Hellenic Endocrine Network. They are unavailable due to copyright and privacy restrictions.

## Supplementary Materials

The following supporting information can be downloaded at https://www.mdpi.com/article/10.3390/nu18030477/s1, Table S1: Loading doses used in each patient subgroup, according to their baseline serum 25-OH vitamin D concentration.
