# Supplementary material for: The Effects of Vitamin D Replacement with a High-Dose Treat-to-Goal Strategy
Source: Nutrients. 2026 Feb 1;18(3):477. doi: 10.3390/nu18030477 (PMC12899947; doi:10.3390/nu18030477)
Supplement: Supplementary file 1 [file nutrients-18-00477-s001.zip › nutrients-4060825-supplementary.pdf]

Table S1: Loading doses used in each patient subgroup, according to their baseline serum 25-OH vitamin D concentration.

Baseline vitamin D (ng/ml)	Loading dose	Loading dose total (over 2 months)
20.0-29.9	25,000 IU once weekly	200,000 IU
10.0-19.9	25,000 IU twice weekly	400,000 IU
<10.0	25,000 IU thrice weekly	600,000 IU
